# Demography and threats to population growth of *Cirsium pitcheri*, a threatened dune plant, in Wisconsin

**DOI:** 10.1002/ece3.10870

**Published:** 2024-02-15

**Authors:** Pati Vitt, E. Binney Girdler, Jeffrey M. Gorra, Tiffany M. Knight, Kayri Havens

**Affiliations:** ^1^ Department of Natural Resources Lake County Forest Preserve District Libertyville Illinois USA; ^2^ Program in Plant Biology and Conservation Northwestern University Evanston Illinois USA; ^3^ Negaunee Institute for Plant Conservation Science and Action, Chicago Botanic Garden Glencoe Illinois USA; ^4^ Department of Biology Kalamazoo College Kalamazoo Michigan USA; ^5^ Department of Community Ecology Helmholtz Centre for Environmental Research – UFZ Halle (Saale) Germany; ^6^ German Centre for Integrative Biodiversity Research (iDiv) Halle‐Jena‐Leipzig Leipzig Germany; ^7^ Institute of Biology Martin Luther University Halle‐Wittenberg Halle (Saale) Germany

**Keywords:** climate change, conservation of rare plant species, *Cirsium pitcheri*, habitat disturbance, Lake Michigan, population ecology, seed predation

## Abstract

Multi‐year and multi‐site demographic data for rare plants allow researchers to observe threats and project population growth rates and thus long‐term persistence of the species, generating knowledge, which allows for effective conservation planning. Demographic studies across more than a decade are extremely rare but allow for the effects of threats to be observed and assessed within the context of interannual environmental variation. We collected demographic data on the Threatened plant *Cirsium pitcheri* in two sites from 2011–2022. These sites were chosen because one exhibited the presence of non‐native seed predators while the other did not, and we hypothesized that we would see declines and potentially extinction of the population threatened by predation. Over the course of our study, we observed additional threats, such as human trampling and high lake levels, which led to significant erosion, sand burial, and storm damage to plants. We find high interannual variation in vital rates and population growth rates for both populations, which mask the overall effects of predation. We observed dramatic declines in plant survivorship and population growth rates in both sites in the years with high lake levels. We conclude that high lake levels, which are expected to become more frequent with climate change, pose a significant threat to all near‐shore populations of *C. pitcheri*.

## Graphical abstract

Populations of *Cirsium pitcheri*, a rare thistle endemic to the Great Lakes of North America, are impacted by external forces, or threats, such as habitat conversion, trampling, and exotic seed‐eating weevils. However, populations in Wisconsin were adversely affected by changing lake levels, with dramatic declines in plant survivorship and population growth rates occurring in years with record or near‐record high lake levels. High lake levels are expected to become more frequent with climate change and pose a significant threat to all near‐shore populations of *C. pitcheri*.

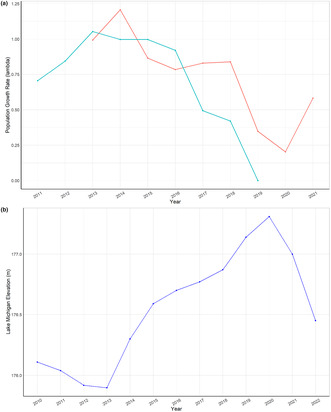

## 1 INTRODUCTION

Understanding the demography and population dynamics of rare plants is crucial for effective conservation and management (Maschinski & Albrecht, 2017; Menges, 2000; Schemske et al., 1994; Silvertown et al., 1996). Rare plants often face numerous threats that can significantly affect their long‐term survival (Corlett, 2016; Ehrlén et al., 2016; Oostermeijer, 2003); however, it can be difficult to identify threats and quantify their effects on populations because they can vary across space and time. Vital rates across the life cycle of a plant, such as survival, growth, and reproduction, determine the dynamics of the population (Caswell, 2001; Ellner et al., 2016). Modeling variation in vital rates across multiple sites or experimental treatments that experience different types and magnitudes of one or more threats is a common approach to discern how individual threats affect populations (Ellner et al., 2016) and can help to identify potential interventions or management strategies that could change the trajectory of a population (Crone et al., 2011). Long‐term demographic data are invaluable for capturing year‐to‐year variations in vital rates, for observing threats that may vary across time, and for quantifying whether sites respond similarly or differently to the same threats across time. However, because collecting field demographic data are a time‐ and labor‐intensive endeavor, a few studies include multiple sites over long‐term time frames (Compagnoni et al., 2021).

Pitcher's Thistle (*Cirsium pitcheri* (Torr. Ex Eaton) Torr. & A. Gray) is endemic to the dune system of the western Great Lakes. This plant is considered a keystone species because it provides critical resources to a wide variety of insect pollinators (Jolls et al., 2019; Sandacz et al., 2023; Vitt et al., 2020). During periods of low lake levels, *C. pitcheri* colonizes the near‐shore habitats in its preferred open sandy habitat, while the species is constrained to the open higher dunes during periods of high lake levels (Loveless, 1984; McEachern, 1992). *Cirsium pitcheri* was federally listed as threatened by the USA in 1988 (USFWS, 1988); the primary threats include habitat loss from conversion of shoreline for recreation and other human uses, invasive species such as *Gypsophila paniculata* L. (baby's breath) and *Centaurea stoebe* L. (spotted knapweed) (Havens et al., 2012), and climate change (Vitt et al., 2010).

In 2010, a new threat—weevil seed predation—was identified for *C. pitcheri* (Havens et al., 2012) by *Larinus* (Phyllonomeus) *carlinae* (Olivier, 1807) (formerly *L. planus*). While *C. pitcheri* is native and rare, there are several *Cirsium* species and close relatives that are exotic and noxious, causing millions of dollars of damage for forage and farmlands. Beginning in the early 1970s, efforts have been underway to identify and release seed predators and other herbivores to control the spread of these species (e.g., Hawkes et al., 1972; Kok & Surles, 1975). Subsequent releases of biocontrol agents targeting spotted knapweed (*Centaurea stoebe*) have included other species of seed‐eating weevils in the genus *Larinus* (e.g., Groppe, 1990). Seed‐eating weevils can significantly reduce seed production of target invasive plants. Unfortunately, seed weevils may also target the reproductive structures of their close relatives resulting in population declines of rare native species (Louda & O'Brien, 2002). At latitudes where the phenology of native thistle populations overlaps with exotic thistles, seed‐eating weevils may preferentially oviposit on the earliest available flowering heads, regardless of species. Although *L. carlinae* prefers *Cirsium arvense* (L.) Scop. to *C. pitcheri* when given a choice (Havens and Vitt, unpublished data), *C. pitcheri* experiences severe damage at many sites presumably due to its earlier flowering phenology. Using matrix population models, weevil seed predation was found to reduce population growth of *C. pitcheri* by 10%–12% and to decrease the time to extinction by 50% to 13 years (Havens et al., 2012).

The new threat of non‐native seed predators motivated us to initiate a long‐term demographic study (2011–2022) at two sites with *C. pitcheri* in Door County, Wisconsin. Based on the results of Havens et al. (2012), we hypothesized that the population at Whitefish Dunes State Park, which has weevils, would decline through time, predominantly because of low recruitment, and would potentially reach extinction within the time frame of our study. We also hypothesized that the nearby population at Ship Canal Nature Preserve, in which weevils were not present, would remain stable over the same period. By studying two populations for many years, we also aimed to document the existence and temporal nature of other threats to *C. pitcheri*. Our study period overlapped with a single oscillation of lake level from a record low to a record high (NOAA, 2023), providing an opportunity to examine the demography of this species throughout a broad range of lake levels. Furthermore, trampling by people is a threat observed in other populations (A. K. McEachern, personal communication).

The shoreline habitat in the Great Lakes of North America is heavily influenced by lake level, which is controlled by the evaporation–precipitation ratio and isostatic rebound (Hansel & Mickelson, 1988; Larsen, 1985) and by eolian processes that sculpt the dunescape (Kilibarda & Shillinglaw, 2015). Although dynamic, historically there has been a relatively regular temporal pattern to lake‐level fluctuations, for instance Lake Michigan levels fluctuate on roughly 30‐, 100‐, and 300‐year cycles (McEachern et al., 1994). The lowest lake levels are associated with less sand movement, relatively stable dunes, and wide beaches. When lake levels are low, the sand that is deposited is blown inland. High lake levels result in narrow beaches and sand accumulation in offshore bars. When lake levels are high, therefore, sand is not available for dune building. Following the period of high lake level, rapid sand accretion occurs inland from the beach as sand moves from the offshore bars back onto the shore (McEachern, 1992). Over the long term, lake levels are projected to be higher as a result of climate change, which will bring higher rates of precipitation to the region (Hayhoe et al., 2010).

The viability of rare plant populations can be influenced by threats, such as habitat loss, climate change, land use change, and invasive species, and by conservation efforts, such as habitat protection, invasive species management, and habitat restoration. Structured population models, such as matrix population models (Caswell, 2001; Salguero‐Gómez et al., 2015) and integral projection models (IPMs), can quantify how such threats and conservation efforts influence the vital rates and long‐term trajectories of populations. IPMs are preferred when the state variables, such as size, are continuously distributed (Ellner et al., 2016; Ellner & Rees, 2006; Levin et al., 2022), as is the case for our study species. Here, we use IPMs to project the population growth rates of two populations of a coastal dune endemic plant that are relatively close geographically, but face a different suite of threats.

## 2 METHODS

### 2.1 Study species

**FIGURE 1:**
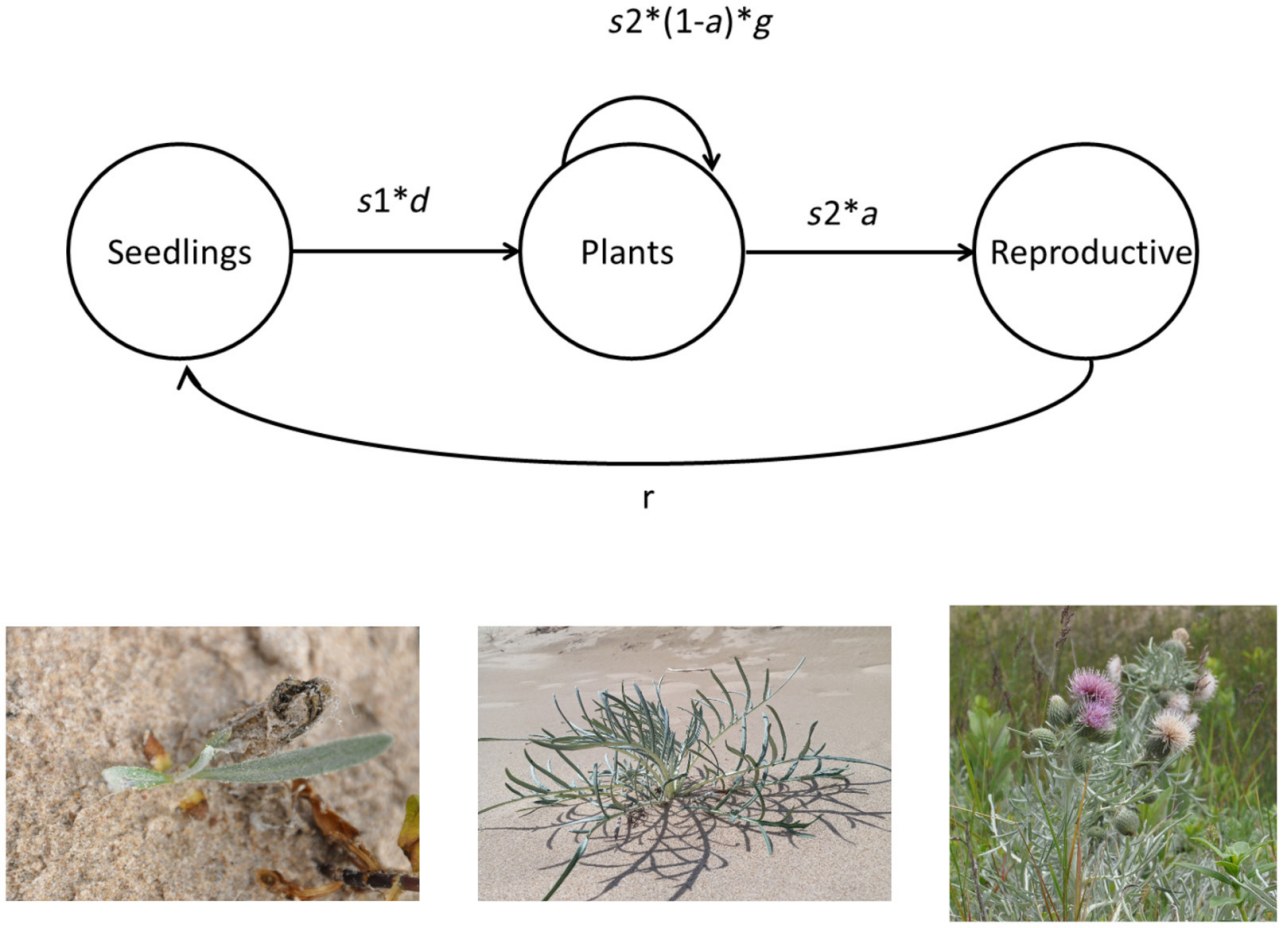
Life cycle of *Cirsium pitcheri* (top) and pictures of each class (bottom). Seedlings survive with probability *s*1 and enter the non‐reproductive class starting at mean size *d*. Non‐reproductive plants survive with probability *s*2, and surviving plants have probability *a* of entering the reproductive class; *g* describes the change in size for those plants that remain in the non‐reproductive class from one year to the next. *r* is the number of seedlings produced per reproductive plant.

*Cirsium pitcheri*, commonly known as Pitcher's or dune thistle, is a native plant that is endemic to the shorelines of the western North American Great Lakes and is listed as threatened by the United States and of Special Concern by Canada. Populations of *C. pitcheri* can be found in Indiana, Michigan, and Wisconsin, on dunes bordering Lakes Michigan, Huron, and Superior. It was extirpated from Illinois in the early 1900s but has been reintroduced at several locations including Illinois Beach State Park. *Cirsium pitcheri* is also present in Canada, including southeastern Lake Huron, Georgian Bay, Manitoulin Island, and Lake Superior (Nantel et al., 2018). This species primarily thrives in open sandy areas that are regularly subjected to natural disturbance processes (Loveless, 1984; McEachern et al., 1994). *Cirsium pitcheri* is a monocarpic perennial (McEachern, 1992) generally living 2–11 years with three life cycle classes: seedlings, non‐reproductive plants, and reproductive plants. Seedlings, easily identifiable by the presence of cotyledons, exist for a single year. Surviving seedlings progress to the non‐reproductive plant class, where they can persist for multiple years before transitioning to the reproductive class. The species is self‐compatible, but florets are protandrous and require pollinator visitation for successful seed set. The species is visited by a wide variety of bees, butterflies, flies, and wasps (Vitt et al., 2020). The life cycle of *C. pitcheri* is shown in Figure 1.

Seeds of *C. pitcheri* are orthodox and dormant when shed (Hamze & Jolls, 2000). Seeds germinate following a period of cold stratification. In the laboratory, additional seedlings flush after each of three successive cold treatments before the seed bank is exhausted (Gijsman & Vitt, 2021; Hamze & Jolls, 2000), and therefore, the species may form an ephemeral soil seed bank in situ (Hamze & Jolls, 2000). Because *C. pitcheri* does not spread vegetatively, recurring establishment by seedling recruitment is required to maintain populations.

### 2.2 Study sites and lake level through time

**FIGURE 2:**
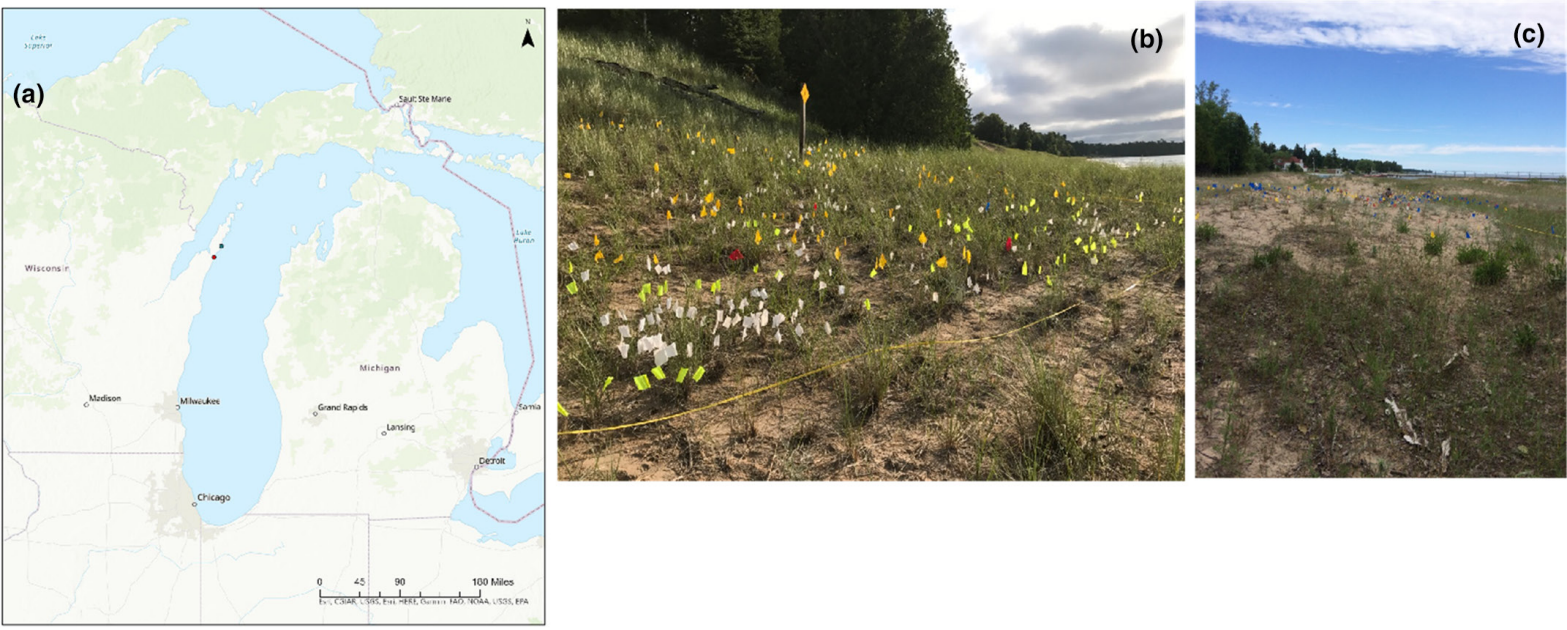
(a) Map showing locations of study sites WFD (blue dot) and SC (red dot). (b) Photograph of WFD in 2016. (c) Photograph of SC in 2016. Pin flags inside plots show locations of individual *C. pitcheri* plants.

This study was conducted at two sites in Door County, Wisconsin, USA: Whitefish Dunes State Park and Ship Canal Nature Preserve (see locations in Figure 2a). The study area at Whitefish Dunes State Park (WFD) consists of a parabolic dune system, stabilized in many areas with large stands of marram grass (*Ammophila breviligulata* Fernald) and relatively few other species in the dunes, although common milkweed (*Asclepias syriaca* L.), beach wormwood (*Artemesia campestris* L.), lyrate rockcress (*Arabis lyrata* L.), and a few other species are present. Behind the dunes, the system transitions into northern mesic forest. Our 10 m by 30 m study plot at WFD was set up in the foredune area, as access to other zones is not possible at this site without harming the habitat due to the steep incline of the back dune area (Figure 2b). Non‐native weevils (*Larinus carlinae*) have been present in this population since at least 2010. These weevils were originally distributed as a biocontrol agent for weedy non‐native thistles but are no longer recommended due to their negative impact on native thistles, including *C. pitcheri*.

Ship Canal Nature Preserve (SC), approximately 24.5 km southwest of WFD, consists of a ridge and swale system where relatively shallow/short sand dunes grade into a forested landscape dominated by pines, hemlocks, and maples. Dune vegetation is more diverse than that at WFD, with several flowering plant species common at this site including *Oligoneruon ohiense* (Frank ex Riddell) G.N. Jones, *Oenothera oakesiana* (A. Gray) J.W. Robbins ex S. Watson & J.M. Coult., *Achillea millefolium* L., *Elymus lanceolatus* (Scribn. & J.G. Sm.) Gould ssp. *psammophilus* (J.M. Gillett & H. Senn) Á. Löve, *Argentina anserina* (L.) Rydb., and *Coreopsis lanceolata* L. Our 10 m by 30 m study plot at SC was placed behind the foredune (Figure 2c). Weevil threats were non‐existent to very minimal at SC throughout this study. Management at this site during our study period has included removal of several large cedar trees (*Thuja occidentalis* L.) in 2016–2017 and ongoing invasive species control that has greatly reduced the cover of these species (primarily *Centaurea stoebe* L., *Sedum acre* L., and *Saponaria officinalis* L.). Managers aimed to open more dune habitat for increased recruitment of *C. pitcheri* seedlings.

Water levels in the Great Lakes vary within and across years. Lake Michigan saw some of its lowest levels in 2012 and 2013 followed by record highs in 2019 and 2020 (NOAA, 2023). Data on water levels in the Lake Michigan/Huron basin were obtained from the GLERL and the U.S. Army Corps of Engineers (https://www.glerl.noaa.gov/data/dashboard/data/levels/1918_PRES/; https://www.lre.usace.army.mil/Missions/Great‐Lakes‐Information/Great‐Lakes‐Information‐2/Water‐Level‐Data/).

### 2.3 Demographic data

Demographic data were gathered annually in the last week of June or first week of July from 2011–2019 at WFD and from 2013–2022 at SC within each 10 m by 30 m plot. To track *C. pitcheri* individuals over time, individuals within the plot were assigned numbered metal ID tags strung on vinyl‐coated wire that were secured to each plant just above the tap root and below the leaves of the rosette.

During the first year of the study at each site, all plants within the plot were tagged (252 plants in 2011 at WFD and 418 at SC in 2013), and their classes and sizes were documented. Plant size was determined by measuring the taproot diameter just below the crown for non‐reproductive plants. Seedlings were not measured, as they vary less in size than non‐reproductive plants and we were concerned that measuring would harm these small individuals. Reproductive plants are also considered a discrete stage class, even though individuals do vary in size. This is because reproductive plants allocate all their below‐ground resources to reproduction, causing taproot size to decrease rapidly across the summer season, and making it difficult to measure and reasonably compare across individuals and years. Our simple consideration of recruitment (number of seedlings per reproductive plant) in the integral projection model matches the data availability, which involved a single snapshot of sampling of seedlings and reproductive plants each year. In the following years, we located previously tagged individuals and recorded their survival status, as well as their class and size (for non‐reproductive plants). Tags were removed from dead plants. New seedlings were tagged each year.

### 2.4 Vital rates across years

We considered six vital rates for *C. pitcheri* (Figure 1): seedling survivorship (*s*1), mean size of new non‐reproductive plants (*d*), survivorship of non‐reproductive plants (*s*2), the proportion of non‐reproductive plants that advance to the reproductive class (*a*), change in size of surviving non‐reproductive plants that remain non‐reproductive (*g*), and seedlings produced per reproductive plant (*r*). We used a linear mixed‐effect model to quantify the variation in *C. pitcheri*'s vital rates across years. We consider the possibility that size will be a main predictor for the vital rates of non‐reproductive plants: *s*2, *g*, and *a*. Size was not considered as a predictor for any other vital rates. Survival (*s*1, *s*2) and probability of flowering (*a*) were modeled as Bernoulli‐distributed processes with logit links, while *d* and *g* were treated as normally distributed variables. At each site, we fit models of each vital rate using a random effect of year (Tables A1 and A2).

Due to limited travel/field time, constraints pursuant to our research permits, and the ethical concerns that would surround any destructive sampling, we restricted our measure of recruitment to a very broad vital rate (seedlings produced per reproductive plant, *r*). We calculated *r* by dividing the number of seedlings present in the plot in year *t*1 divided by the number of reproductive plants present in year *t*0. However, we recognize that multiple vital rates underlie *r*, such as heads per reproductive plant, seeds per head, seed survivorship, germination, and early seedling (pre‐census) survivorship (see Section 4).

To visualize the vital rates, we plotted the predictions of these models for each year and site. For the models that included size, we plotted the vital rate for individuals of a specific size: the mean size, for *s*2 and *g*, and one standard deviation larger than the mean for *a*, as this vital rate is consistently low across sites and years for individuals at the mean size.

### 2.5 Population growth rate across years

We projected long‐term population growth rates of sites and years using an integral projection model (IPM, Ellner et al., 2016). We constructed the IPM using the parameters of the vital rates models described above for each site and year. The change in number of non‐reproductive plants from one year to the next (from *t*0 to *t*1) is described by:

$$N\left(z^{\prime },t1\right)=S\left(t0\right)s1d\left(z^{\prime }\right)+\int_{L}^{U}N\left(z,t0\right)s2\left(z\right)\left(1‐a(z)\right)g\left(z^{\prime },z\right)dz$$

The vector *N*(*z*′, *t*1) is the number of non‐reproductive plants at size *z*′ at time *t*1. The first term represents seedlings entering the non‐reproductive plant class: *S*(*t*0) is the number of seedlings in the population at time *t*0, *s*1 is seedling survivorship, and d(*z*′) is the size distribution of new non‐reproductive plants. The second term describes the number and size of non‐reproductive plants that remain in the non‐reproductive class from *t*0, *N*(*z*, *t*0) to *t*1, *N*(*z*′, *t*1). It is defined as an integral with an upper limit U, which is the biggest size observed and a lower limit L, which is the smallest size observed in our study. Both U and L limits were extended to 20% of their values to set upper and lower size boundaries in the model. The integrals were evaluated across 200 equally spaced size bins using the midpoint rule. The third term describes the loss of individuals from the non‐reproductive plant class from t0 to t1 because they enter the reproductive class.

The number of seedlings in the population in t1 is described by:

$$S\left(t1\right)=A\left(t0\right)r$$

where *A*(*t*0) is the number of reproductive adults present in *t*0 and *r* is the seedlings produced per reproductive adult.

Finally, the number of adults the population in *t*1 is described by:

$$A\left(t1\right)=\int_{L}^{U}N\left(z,t0\right)s2\left(z\right)a\left(z\right)dz$$

We implemented these IPMs using the ipmr package in R (Levin et al., 2021) and used the IPMs to calculate population growth rate (*λ*) for each site and year.

### 2.6 Population growth rate with and without weevils

We quantified the effects of seed‐eating weevils on population growth rate by altering parameter *r* in the IPM. On average, weevil presence reduces seed production per head by 60% (Gijsman et al., 2020). For the SC population, where weevils are not present, we decreased *r* by 60% and projected *λ* for each year to quantify what *λ* would be if weevils were present in the population. The median and variance of *λ* across years with and without weevils was quantified for the population and visualized using boxplots. Likewise, for WFD, where weevils are present, we increased *r* by 60% to quantify what *λ* would be if weevils were absent.

## 3 RESULTS

### 3.1 Observed threats through time

**FIGURE 3:**
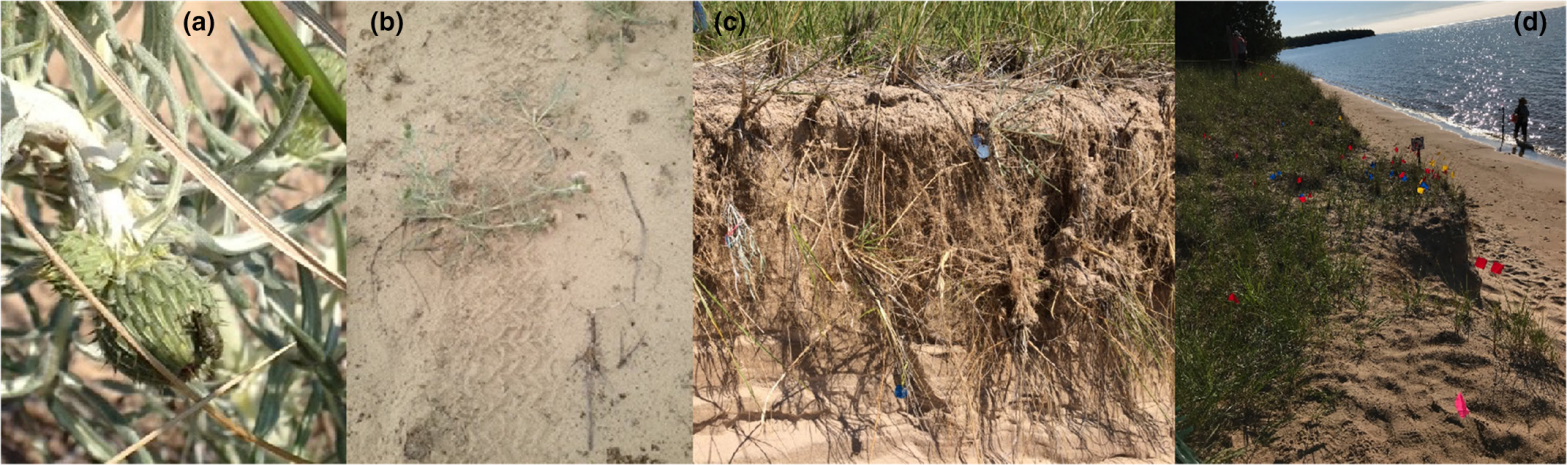
Threats to *C. pitcheri* observed at WFD and SC. (a) Seed weevils (*L. carlinae*) on a *C. pitcheri* flowering head at WFD. (b) Observed damage from ATVs at SC in 2013. (c) Beginning of the loss of the foredune at WFD in 2018 due to rising lake levels. In this photo, the tags of plants are visible as plants perish due to the loss of the foredune. (d) This photo shows P. Vitt standing at WFD in 2018 at the GPS location that represents the edge of the original plot. The foredune and all plants in the plot were lost due to the high lake levels.

We set up these populations to differ in a single threat: the presence of seed weevils (Figure 3a). However, our observations through time identified other threats to *C. pitcheri* at these two sites. First, we observed damage on many plants at SC in 2013 from All Terrain Vehicles (ATVs) (Figure 3b). Second, we observed significant human trampling in WFD in 2018. Both of these events caused observable plant death. Most prominently, we observed rising lake levels over the course of our study. These resulted in the complete loss of the WFD foredune and the loss of all plants in our plot by 2019 (Figure 3c,d). In SC, the plot was located behind the foredune and should have been more protected from rising lake levels. However, the invasive plant management that occurred at this site had the counterproductive consequence of exposing plants in our SC plot to the effects of sand movement, exacerbated by winter storms and changing lake levels, resulting in high mortality of plants in the years following peak lake level.

### 3.2 Vital rates across years

**FIGURE 4:**
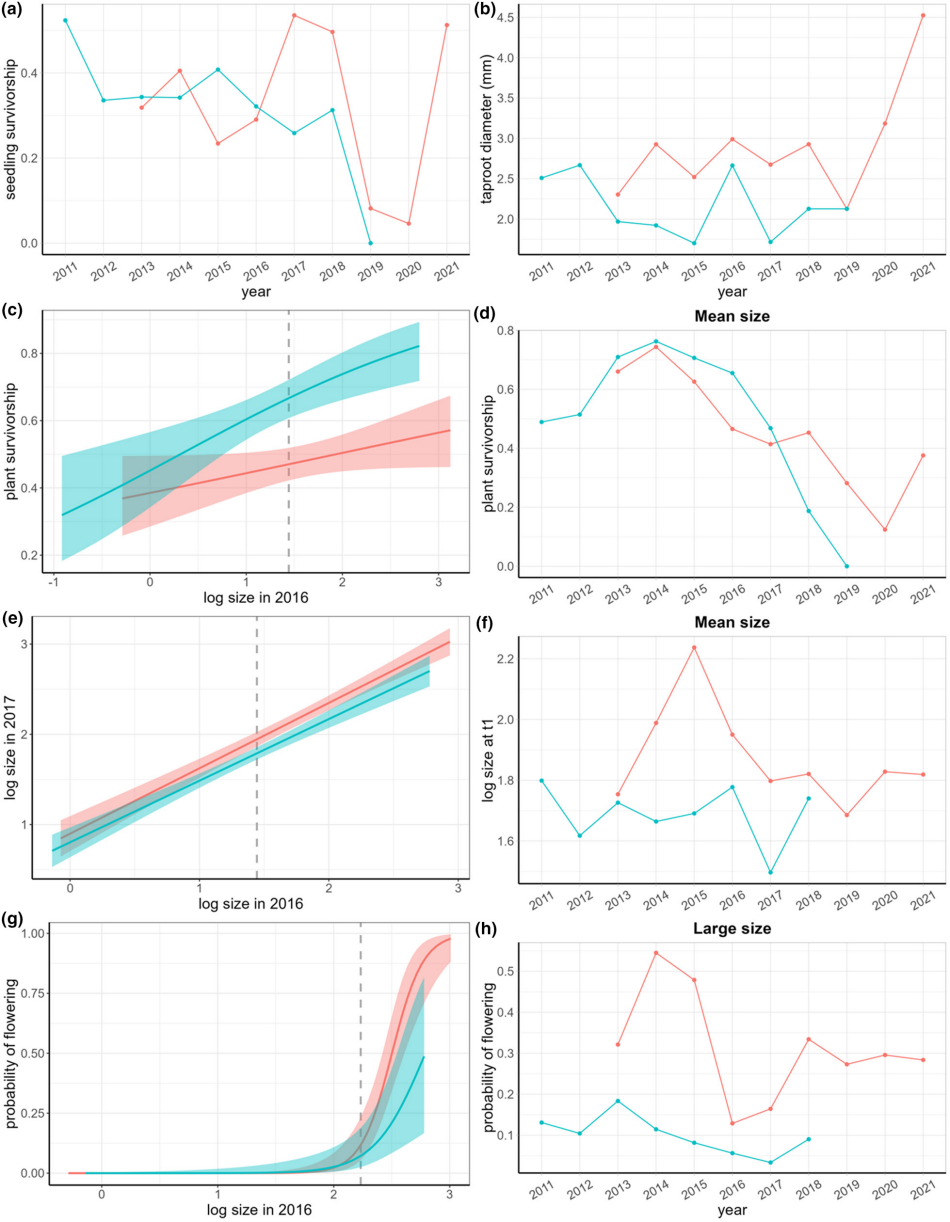
Demographic vital rates through time of *C. pitcheri* at WFD (blue) and SC (red). (a) Mean survivorship of seedlings. (b) Mean size (taproot diameter in mm) of new non‐reproductive plants (individuals were seedlings in the previous year). (c) Relationship between non‐reproductive plant size in *t*0 and survivorship in *t*1 shown for a single year (2016) for each site; plants that are larger in *t*0 are more likely to survive; shaded areas represent standard error (SE). The dashed gray line shows the mean size of non‐reproductive plants. (d) Survivorship of plants at the mean non‐reproductive plant size for each site and year. (e) Relationship between size *t*0 and size *t*1 of non‐reproductive plants shown for a single transition (2016–2017); shaded area = SE. The dashed gray line shows the mean size of non‐reproductive plants. (f) Size at *t*1 of non‐reproductive plants that were the mean size in *t*0 for each site and year. (g) Relationship between the size of non‐reproductive plants in *t*0 and their probability of flowering in *t*1; larger plants are more likely to flower in the next year. Shaded area = SE. The dashed gray line shows the mean size plus one standard deviation. (h) Probability of flowering for non‐reproductive plants at the mean plus one standard deviation of size in t0 for each site and year.

Seedling survivorship, *s*1, was highly variable across populations and time (Figure 4a), ranging from no survivorship (WFD in 2019) to half of the seedlings surviving from one year to the next (WFD in 2011, SC in 2017). The size of these surviving seedlings when they first entered the new plant stage, *d*, was similar across time, generally between 2.0 and 3.0 mm crown diameter (Figure 4b). Survivorship of non‐reproductive plants, *s*2, depended on plant size in *t*0 (Figure 4c, Tables A1 and A2) and was variable and showed a decreasing trend from 2015–2020 (Figure 4d). The size of non‐reproductive plants in *t*1 depended on plant size in *t*0 (Figure 4e) and was highly variable across years (Figure 4f). Larger non‐reproductive plants in *t*0 were more likely to advance to the reproductive class in *t*1 (Figure 4g). The probability of flowering, *a*, was also highly variable across time (Figure 4h). Recruitment of new seedlings into the population, *r*, was not lower in WFD than SC as was expected due to the presence of seed‐eating weevils. Both populations show high interannual variation in recruitment ranging from less than one seedling produced per reproductive plant to more than 27 (Table 1).

**TABLE 1:** Recruitment of *C. pitcheri* at WFD and SC across years. Recruitment (*r*) is calculated based on the quotient of the number of seedlings at time *t*1 and the number of reproductive plants at time *t*0.

Site	Year *t*0	Number of reproductive plants *t*0	Number of new seedlings *t*1	Recruitment (*r*)
SC	2013	28	477	17.04
SC	2014	23	303	13.17
SC	2015	38	275	7.24
SC	2016	50	602	12.04
SC	2017	37	640	17.30
SC	2018	28	280	10.00
SC	2019	27	113	4.19
SC	2020	16	59	3.69
SC	2021	3	NA	NA
WFD	2011	21	82	3.90
WFD	2012	13	359	27.62
WFD	2013	8	191	23.88
WFD	2014	7	121	17.29
WFD	2015	6	143	23.83
WFD	2016	7	101	14.43
WFD	2017	5	4	0.80
WFD	2018	4	48	12.00
WFD	2019	7	NA	NA

### 3.3 Population growth rate across years

**FIGURE 5:**
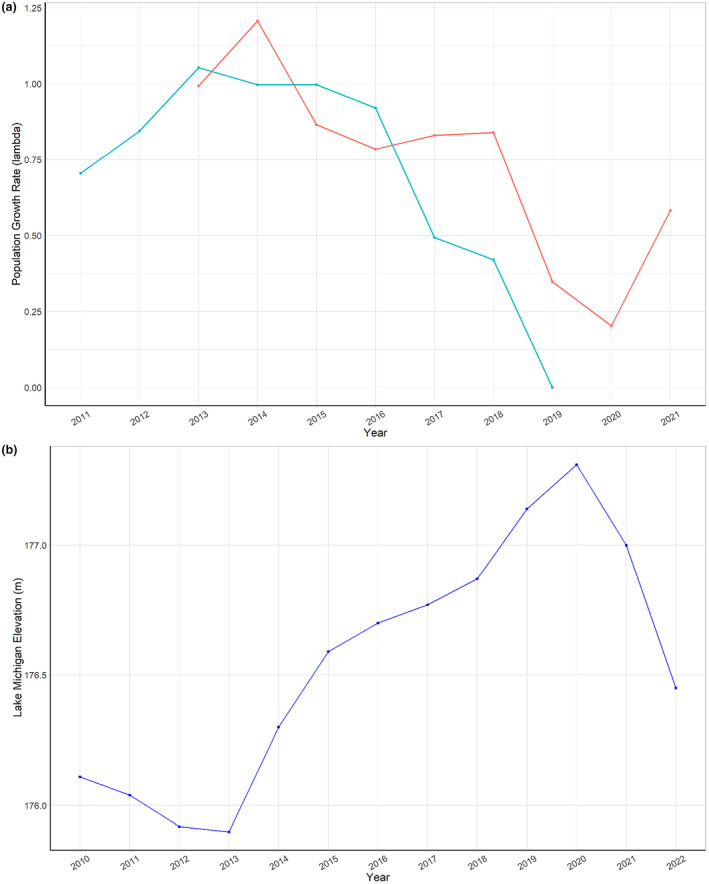
(a) Population growth rate (λ) across years for WFD (blue) and SC (red), where *λ* > 1 indicates a growing population. (b) Elevation of Lake Michigan (m above sea level) during our study period.

For both populations, we see very few years where populations are growing (*λ* > 1), and we see very low *λ* in the later years of the study (Figure 5a). The declining trend in λ through time corresponds inversely with increasing lake levels (Figure 5b).

### 3.4 Population growth rate with and without weevils

**FIGURE 6:**
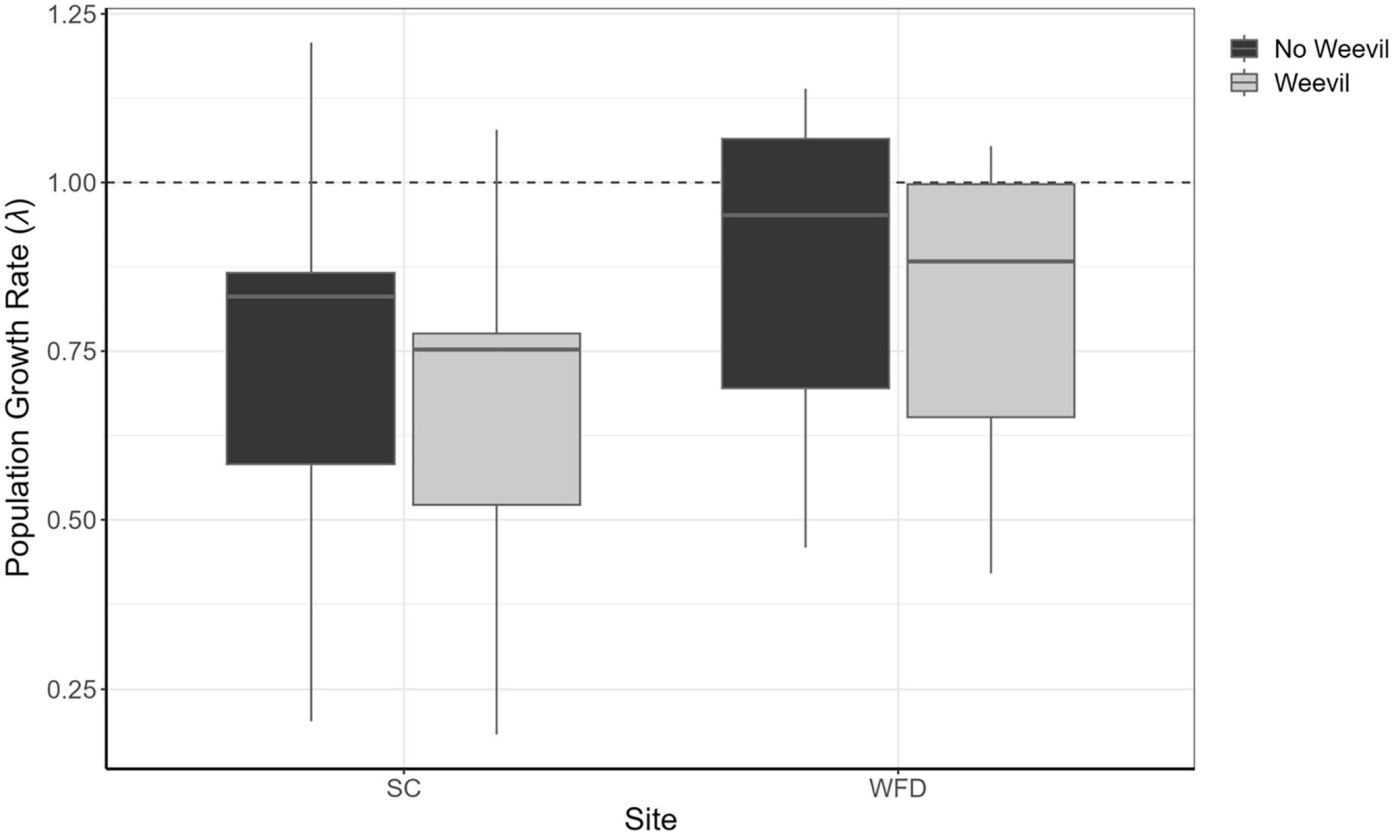
Box plot showing median population growth rate (*λ*) and the first and third quartiles of *λ* for WFD and SC modeled with a discounted recruitment rate (−60%) to simulate the effect of weevils where they do not occur (SC) or with an enhanced recruitment rate (+60%) to simulate the effect of no weevils (WFD). *λ* = 1 (dashed line) indicates a stable population.

The effects of weevil predation on population growth rate (*λ*) are small relative to the interannual variation, which results in large variation in *λ* for both populations across the study period (Figure 6).

## 4 DISCUSSION

This study was initiated to compare populations of *C. pitcheri* with and without seed weevils, a threat with the potential to reduce population time to extinction to just a few years, depending upon the size and stability of the site and the presence of additional threats (Havens et al., 2012). We hypothesized that Whitefish Dunes State Park, Door County, WI, a site with seed weevils, would decline through time, while the population at Ship Canal Nature Preserve, Door County, WI, would remain stable or grow. We collected long‐term data at these sites across a period of fluctuating lake levels and have found that our long‐term demographic data show that volatile lake levels are a more important and more rapidly acting threat. Our results show overall declines for the two WI populations of *C. pitcheri* and identify threats to the viability of populations of this species, ranging from direct human damage (trampling by foot and ATVs), weevil seed predation, and high lake levels. These threats are likely not independent. For example, the high human trampling we observed in 2018 was due to recreational visitors to the dunes taking new routes to the shoreline in response to the high lake levels.

### 4.1 Local‐scale factors affecting population dynamics

*Larinus carlinae* has been observed to reduce the seed set of *C. pitcheri* by 60% (Gijsman et al., 2020). In addition, this seed‐eating weevil also significantly reduced seed mass, which is positively associated with germination probability in this species (Hamze & Jolls, 2000; Gijsman & Vitt, 2021) because larger seeds are more likely to emerge especially from areas with deep seed burial depth (Hamze & Jolls, 2000). When grown in a common garden, plants from our two sites (WFD and SC) were similar in their size and seed set; however, plants from SC produced seeds with higher mass than those from WFD (Gijsman et al., 2020). In the field, plants growing at WFD produced heavier seed than those growing at SC (Gijsman et al., 2020).

We expected the reduced fecundity at WFD (due to the presence of weevils) to significantly reduce recruitment. However, we found that recruitment was similar across the two sites, despite high densities of weevils and heavy damage observed at WFD (personal observations). Given that we know heavy seed losses occurred in WFD due to weevils (Gijsman et al., 2020), there must be other factors at WFD that explain the high number of seedlings produced per reproductive plant at this site compared to SC, such as more heads per reproductive plant, more seeds per head, heavier seeds, higher post‐dispersal seed survivorship, higher germination rates, and/or higher early (pre‐census) seedling survivorship. Dune structure appears to be more stable at WFD, largely as a result of a higher presence of *Ammophila breviligulata*, which acts to reduce sand movement. As the seeds at WFD are also heavier overall than those produced at SC (Gijsman et al., 2020), site conditions might be more conducive to higher establishment rates, absent the effects of lake level. In the future, more detailed research is needed that focuses on capturing the vital rates within the recruitment process would provide further mechanistic insight between these sites.

### 4.2 Broad‐scale factors affecting population dynamics

The long‐term average level for Lake Michigan is 176.4 m with a high of 177.5 m and a low of 175.6 m (NOAA, 2023). Historically, lake levels fluctuate in cycles in response to climate, especially precipitation (Gronewold et al., 2013), typically as much as +/−lm around the mean high‐water level on 20‐ to 30‐year cycles and can fluctuate ~0.5 m annually (McEachern, 1992, and references therein). Precipitation in the Lake Michigan basin recorded the highest average annual levels for which data are publicly available in 2019, with correspondingly high lake levels in June 2020 of 177.45 m, with a low of 175.57 m in January of 2013 (NOAA, 2023). Thus, data collected during our study capture the correlated population dynamics of an unusual lake oscillation from historic lows to historic highs in less than a decade. While unusual, this rapid and dramatic lake oscillation might be a more common phenomenon in the future with changing patterns of precipitation projected as our climate changes in response to human‐induced carbon release. For example, Kayastha et al. (2022) report that the Lake Michigan–Huron basin is projected to experience an average annual increase in water level of +0.44 m by 2040–2049.

*Cirsium pitcheri* is at risk of rapid population declines if lake levels continue to change as rapidly and drastically as they have in recent years. Historically, this species could persist and even thrive in a dynamic dune system by recolonizing new open sandy habitats when they became available and persisting in backdune habitats during years with high lake levels. However, it is likely that the current situation, which involves smaller and more fragmented populations combined with more rapid and dramatic lake fluctuations, as well as more severe winter storms, creates a situation in which metapopulation dynamics cannot allow landscape‐scale persistence. Our study populations appear to have only local linkages with other subpopulations along a narrow shoreline. These other subpopulations could act as either sources or sinks; however, spatially autocorrelated responses to climate and depressed seed production due to weevils might limit the potential for source populations to rescue sinks. Thus, it appears that, although these sites may once have behaved as metapopulations, currently this is not the case as the patterns we have observed violate the conditions for a metapopulation described by McEachern et al. (1994). Ongoing conservation efforts for this species must, therefore, be analyzed at a much larger spatial scale to fully capture the potential for recovery, if this is even possible in the current era of climate extremes.

### 4.3 Generalizability and future monitoring

Our study is limited in its spatial extent, focusing on two populations that are in close proximity to each other. While more studies are needed to understand the generalizability of our results, we posit that all near‐shore populations might be at risk during periods of high lake levels or during the period just after when sand is blown inland (McEachern, 1992). Indeed, in another study on long‐term *C. pitcheri* demography in Michigan, Girdler et al. (in revision) found that location on the dune influences the direction and magnitude in which plants respond to lake levels and precipitation variation through time. Plants on the foredune respond negatively to increasing precipitation, likely due to the direct harm and sand burial they receive from winter storms. Plants in the backdune responded positively to increasing precipitation, likely because they were protected from storm damage and were able to capitalize on the increased water availability in those years. Together, our results and those of Girdler et al. (unpublished data, E.B.G., T.M.K., S.M.E., A.C., R.L., J.E.M., S.I.H., C.L.J.) suggest that future monitoring and management of *C. pitcheri* in the face of climate change must consider dune location.

Our results suggest that monitoring of *C. pitcheri* is best accomplished through surveys that are rapid but broad in their spatial extent in combination with surveys that provide detailed demography. For the broad surveys, counts of reproductive plants are the only option, as other count methods are laborious and potentially inaccurate due to the cryptic nature of individual plants. These counts could provide a rapid way to quantify the changing patterns of population size and spatial distribution, including localized extinction and colonization dynamics. However, it is unproven how well these counts are correlated with trends from more detailed demographic studies. Detailed demographic studies in the future should implement plots that run perpendicular to the lake, to capture the effects of dune location on responses to threats such as rising lake levels (unpublished data, E.B.G., T.M.K., S.M.E., A.C., R.L., J.E.M., S.I.H., C.L.J.).

### 4.4 Management and conservation recommendations

Given the threats we observed, some of which are difficult or impossible to manage, we must think about productive avenues forward to recover this threatened species. We recommend a stronger emphasis on assisted migration to more spatially secure sites without immediate human disturbances such as trampling and or habitat conversion. For example, there are several potential sites on the smaller uninhabited islands in the Beaver Island and Potawatomi Archipelagos in Lake Michigan. Islands are ideal as many currently do not have *Larinu*s *carlinae*, and these weevils are not expected to easily spread to these locations. At sites where *C. pitcheri* is already present, restoring the populations on open sites and blowouts in the back dunes has the potential to secure the species in sites to which they might already be locally adapted. We recommend that a range‐wide inventory be conducted whereby the conservation status of this species should only be based upon population size estimates in the rear‐ward side of the dunes as near‐shore populations have proven to be too ephemeral to be considered secure. In addition, we recommend expanding ex situ conservation efforts to ensure that the range‐wide genetic variability (Fant et al., 2013) is present in seed banks. Finally, it is notable that the surveys and subsequent policy decisions on the listing status of *C. pitcheri* were taken by the United States (USFWS) and Canada (COSEWIC, 2010) during a period of low lake levels and did not include information on the threat of introduced weevils. Based on the threats and rapid declines we report here, along with similar results from other recent studies, we recommend that *C. pitcheri* be uplisted to Endangered.

## AUTHOR CONTRIBUTIONS

**Pati Vitt:** Conceptualization (lead); data curation (lead); formal analysis (equal); investigation (equal); methodology (lead); project administration (supporting); supervision (equal); validation (lead); writing – original draft (lead). **E. Binney Girdler:** Formal analysis (lead); software (equal); writing – review and editing (supporting). **Jeffrey M. Gorra:** Data curation (supporting); investigation (supporting); project administration (supporting); supervision (supporting); writing – review and editing (supporting). **Tiffany M. Knight:** Formal analysis (equal); investigation (equal); software (lead); validation (equal); visualization (equal); writing – original draft (equal); writing – review and editing (equal). **Kayri Havens:** Conceptualization (equal); funding acquisition (lead); project administration (lead); supervision (equal); writing – review and editing (equal).

## CONFLICT OF INTEREST STATEMENT

The authors declare no conflict of interest.

## Data Availability

Our long‐term data on *Cirsium pitcheri* demography at WFD and SC are publicly available on Figshare: https://10.6084/m9.figshare.24079287.

## APPENDIX 1

**TABLE A1:** Ship Canal models.

Vital rate (sample size)	Model specification	Parameter estimates				AIC
		Estimate (for fixed effects) or variance (for random year effects)	SE	*z* Value	Pr(>|*z*|)	
*s*1 (*n* = 1397)	surv_t1 ~ (1|year_t0), family = binomial					1711.5
	year_t0 (random)	1.268				
	Intercept (fixed)	−0.9644	0.3915	−2.463	.0138	
*d* (*n* = 346)	ln_diam_t1 ~ (1|year_t0), family = gaussian					534.73
	year_t0 (random)	0.0626				
	Intercept (fixed)	1.046	0.0951	10.99077	<.0001	
*s*2 (*n* = 2481)	surv_t1 ~ ln_diam_t0 + (1|year_t0), family = binomial					3120.45
	year_t0 (random)	0.7683				
	Intercept (fixed)	−0.5755	0.3118	−1.846	.0649	
	ln_diam_t0 (fixed)	0.2675	0.0644	4.153	<.0001	
	*surv_t1 ~ ln_diam_t0 + (ln_diam_t0|year_t0), family = binomial*					*3119.86*
	*surv_t1 ~ ln_diam_t0 + (0 + ln_diam_t0|year_t0), family = binomial*					*3148.54*
*g* (*n* = 995)	ln_diam_t1 ~ ln_diam_t0 + (1|year_t0), family = gaussian					1304.81
	year_t0 (random)	0.1732				
	Intercept (fixed)	0.8741	0.0694	12.592	<.0001	
	ln_diam_t0 (fixed)	0.6935	0.0224	30.954	<.0001	
	*ln_diam_t1 ~ ln_diam_t0 + (ln_diam_t0 |year_t0), family = gaussian*					*1308.51*
	*ln_diam_t1 ~ ln_diam_t0 + (0+ ln_diam_t0|year_t0), family = gaussian*					*1324.28*
*a* (*n* = 1165)	prob_flower ~ ln_diam_t0 + (1|year_t0), family = binomial					578.68
	year_t0 (random)	0.5099				
	Intercept (fixed)	−9.5652	0.6912	−13.84	<.0001	
	ln_diam_t0 (fixed)	4.1706	0.3003	13.89	<.0001	
	*prob_flower ~ ln_diam_t0 + (ln_diam_t0|year_t0), family = binomial*					*582.20*
	*prob_flower ~ ln_diam_t0 + (0 + ln_diam_t0|year_t0), family = binomial*					*581.40*
*r* (*n* = 9 years)	Recruitment = num_seedlings_t1/num_flowering_t0					NA

*Note*: Seedlings survive with probability *s*1 and enter the non‐reproductive class starting at mean size *d*. Non‐reproductive plants survive with probability *s*2, and surviving plants have probability *a* of entering the reproductive class; *g* describes the change in size for those plants that remain in the non‐reproductive class from one year to the next. *r* is the number of seedlings produced per reproductive plant. Parameter estimates shown for best models as determined by AIC comparison (if dAIC < 2 the simplest model was chosen). Poorer models are italicized. Recruitment (seedlings per flowering plant) was not a model but a simple calculation. For completeness, AIC is given for all vital rate models even when there was no model selection (e.g., *s*1 and *d*).

**TABLE A2:** Whitefish Dune models.

Vital rate (sample size)	Model specification	Parameter estimates				AIC
		Estimate (for fixed effects) or Variance (for random year effects)	Std. error	*z* Value	Pr(>|*z*|)	
*s*1 (*n* = 1089)	surv_t1 ~ (1 | year_t0), family = binomial					1407.8
	year_t0 (random)	0.1426				
	Intercept (fixed)	−0.6097	0.1588	−3.84	.00012	
*d* (*n* = 271)	ln_diam_t1 ~ (1 | year_t0), family = gaussian					370.06
	year_t0 (random)	0.04609				
	Intercept (fixed)	0.7551	0.0868	8.6963	<.0001	
*s*2 (*n* = 1492)	*surv_t1 ~ ln_diam_t0 + (1|year_t0), family = binomial*					*1784.33*
	surv_t1 ~ ln_diam_t0 + (ln_diam_t0|year_t0), family = binomial					1781.90
	year_t0 (random)	1.0970				
	Intercept (fixed)	−0.8379	0.4014	−2.087	.0369	
	ln_diam_t0 (fixed)	0.4361	0.2227	1.959	.0501	
	ln_diam_t0|year_t0 (random)	0.3064				
	*surv_t1 ~ ln_diam_t0 + (0 + ln_diam_t0|year_t0), family = binomial*					*1796.02*
*g* (*n* = 650)	*ln_diam_t1 ~ ln_diam_t0 + (1|year_t0), family = gaussian*					*867.95*
	ln_diam_t1 ~ ln_diam_t0 + (ln_diam_t0|year_t0), family = gaussian					858.16
	year_t0 (random)	0.1000				
	Intercept (fixed)	0.8634	0.1258	6.8612	<.0001	
	ln_diam_t0 (fixed)	0.5771	0.0784	7.3634	<.0001	
	ln_diam_t0|year_t0 (random)	0.0398				
	*ln_diam_t1 ~ ln_diam_t0 + (0+ ln_diam_t0|year_t0), family = gaussian*					*866.69*
*a* (*n* = 778)	prob_flower ~ ln_diam_t0 + (1|year_t0), family = binomial					234.77
	year_t0 (random)	0.4672				
	Intercept (fixed)	−10.091	1.135	−8.890	<.0001	
	ln_diam_t0 (fixed)	3.736	0.484	7.719	<.0001	
	*prob_flower ~ ln_diam_t0 + (ln_diam_t0|year_t0), family = binomial*					*NA – failed to converge*
	*prob_flower ~ ln_diam_t0 + (0 + ln_diam_t0|year_t0), family = binomial*					*235.14*
*r* (*n* = 9 years)	Recruitment = num_seedlings_t1/ num_flowering_t0					NA

*Note*: Seedlings survive with probability *s*1 and enter the non‐reproductive class starting at mean size *d*. Non‐reproductive plants survive with probability *s*2, and surviving plants have probability *a* of entering the reproductive class; *g* describes the change in size for those plants that remain in the non‐reproductive class from one year to the next. *r* is the number of seedlings produced per reproductive plant. Parameter estimates shown for best models as determined by AIC comparison (if dAIC < 2 the simplest model was chosen). Poorer models are italicized. Recruitment (seedlings per flowering plant) was not a model but a simple calculation. For completeness, AIC is given for all vital rate models even when there was no model selection.
